# Hospital Meetings

**Published:** 1895-11-30

**Authors:** 


### HOSPITAL MEETINGS.

### HOSPITAL SUNDAY FUND.

A meeting of the council of this institution was held at the Mansion House yesterday afternoon, to determine the report for the year and to transact the current business. The present Lord Mayor presided for the first time. The report of the year showed that the fund amounted to £60,360, being the highest since the foundation in 1873.

We abstract the following passages from the report of the Council for the year ending 31st October, 1895:—

The Council desire to place on record the indebtedness they feel to the late Lord Mayor, Sir Joseph Renals, Bart., who has displayed a kind interest in the success of the Hospital Sunday Fund during his mayoralty, and to Mr. W. J. Soulsby, who for twenty years has been private secretary to successive Lord Mayors.

Amongst the preachers who specially exerted themselves and so materially contributed to the splendid results of 1895 mention must be made of the special sermons preached by the Archbishop of Canterbury; the Chief Rabbi; the Dean of Westminster, Dean Farrar; the Archdeacon of London, Canon Sinclair; Canons Ainger, Fleming, Wilberforce, and Keatinge; the Rev. C. J. Ridgeway, and many others who united with the Press to raise the £60,000 collected.

The Editors of the Metropolitan Press enthusiastically co-operated, and Mr. Arthur Walter, proprietor of the *Times*, for the first time in its history, consented to insert and give prominence to the blocks which they republished from the special supplement of The Hospital, nearly 60,000 copies of which were circulated at the cost of a few friends of the Editor, and £18,000 was sent in contributions to the Lord Mayor as the result of The Hospital appeal. The *Lancet* issued once more a special supplement, which was widely circulated, and the editors of the *Times, Standard, Daily News, Daily Chronicle, Morning Post, Daily Graphic, Morning Advertiser*, the *Pall Mall, St. James's*, and *Westminster Gazettes*, the *Globe*, and the Sunday and weekly papers all rendered willing assistance, with results so fruitful that the Council hope the Press may be encouraged to continue similar efforts year by year, and so to provide that the Metropolitan Hospital Sunday Fund shall never again yield less than £50,000 for the metropolitan medical charities.

The Lord Mayor said he was gratified to find that the amount realised this year had reached so large a sum. He trusted that during the coming year all would assist him, and he would do all he possibly could to maintain those figures at least. (Hear, hear.) The following gentlemen were elected to fill vacancies on the Council caused by death or retirement: The Rev. Canon Grahame, of St. Mary's College, Brook Green, in place of Canon Gilbert; Rev. D. Anderson, Rev. M. R. Neligan, Rev. H. Russell Wakefield; the Earl of Stamford in place of Lord Sandhurst, retired; Mr. Alderman Faudel Philips—who, it was stated, will probably be next Lord Mayor—in place of Alderman Lusk, retired; Sir Joseph Renals in place of Sir Francis Wyatt Truscott, deceased; and Mr. John Astley Bloxham, senior surgeon of Charing Cross Hospital. Wednesday, December 18th, was fixed for the annual meeting, and Sunday, June 14th next, was determined upon as Hospital Sunday for 1896. Mr. R. B. D. Acland drew attention to the position of St. Thomas's Hospital, and intimated his intention to move an instruction to the Council to include thst hospital in the list of those recommended to receive an award. Mr. Custance, secretary, remarked that in reply to a letter on the subject he had stated that St. Thomas's should show three years' accounts to the Committee of Distribution, and show how they were constituted. There would then be sufficient time to attend to their case. Mr. Acland wished to know whether an annual grant was made to Guy's Hospital under the present system, and if not he would like to make a suggestion that the rules should be altered to enable that to be done. Dr. Marks observed that if these two hospitals would abide by the rules there would be no difficulty whatever. Mr. H. C. Burdett said the Fund was established to support voluntary hospitals as compared with hospitals which had large revenues, and which had been supported out of those revenues. It seemed hard that the Fund should be asked to assist those hospitals, and that it should partially set aside the claims of 107 hospitals because two which had lsrge funds were not able to fill all the beds out of the present endowment. Mr. Acland gave notice of motion on the subject for a meeting of the Council to be specially summoned.

On the motion of the Chief Rabbi, a vote of thanks to the Lord Mayor concluded the proceedings. Among those present at the meeting were the Rev. Dr. Rigg, Archdeacon Sinclair, Sir Stuart Knill, Dr. J. G. Glover, and Mr. R. B. Martin.

---

On Tuesday last the Duke of Devonshire opened the new home at the Colony for Epileptics at Chalfont St. Peter. Mr. Passmore Edwards provided the site some time ago, and has borne the expense of the home also. A full description of the ceremony of Tuesday, and a description of the building, together with an account of the work done up to the present time, will appear in our columns next week.

